# Design of a Diagnostic Immunoassay for Aflatoxin M1 Based on a Plant-Produced Antibody

**DOI:** 10.3390/toxins14120851

**Published:** 2022-12-03

**Authors:** Cristina Capodicasa, Erica Bastiani, Thea Serra, Laura Anfossi, Marcello Catellani

**Affiliations:** 1 Laboratory Biotechnologies, Department of Sustainability, ENEA, Casaccia Research Center, Via Anguillarese 301, 00123 Rome, Italy; 2 Euroclone S.p.A., Via Figino 20/22, 20016 Milano, Italy; 3 Department of Chemistry, University of Turin, Via Giuria 7, 10125 Turin, Italy; 4 Laboratory Bio-Products and Bio-Processes, Department of Sustainability, ENEA, Casaccia Research Center, Via Anguillarese 301, 00123 Rome, Italy

**Keywords:** aflatoxin M1, plant molecular farming, ELISA, recombinant antibody, *Nicotiana benthamiana*, transient expression, food safety

## Abstract

A new green competitive ELISA for aflatoxin M1 quantification in raw milk was developed. This diagnostic tool is based on an anti AFM1 mAb produced by plant molecular farming in alternative to classical systems. Our assay, showing an IC_50_ below 25 ng/L, fits with the requirements of EU legislation limits for AFM1 (50 ng/L). Optimal accuracy was achieved in correspondence of the decision levels (25 and 50 ng/L), and the assay enabled AFM1 quantification in the range 5–110 ng/L, with limit of detection 3 ng/L. Moreover, to evaluate a real applicability in diagnostics, raw milk-spiked samples were analysed, achieving satisfactory recovery rates of AFM1. In conclusion, an efficient and ready-to-use diagnostic assay for the quantification of aflatoxin M1 in milk, based on a plant-produced recombinant mAb, has been successfully developed.

## 1. Introduction

Mycotoxins are small organic molecules produced, in appropriate microclimatic conditions, by several fungi contaminating a wide range of important raw food commodities (cereals, legumes, spices, and nuts). The Food and Agriculture Organization (FAO) estimates that 25% of the world-wide production is affected by mycotoxins; among these, many are represented by aflatoxins. Even if mycotoxins usually do not cause an acute direct adverse effect, a chronic exposure to these contaminants may pose a significant risk to consumers [1]. Aflatoxin B1 (AFB1) is the most potent natural carcinogen known and has been classified by the International Agency for Research on Cancer (IARC) as carcinogenic to humans (Group 1) [2,3]. Carcinogenic activity of aflatoxin is attributed to covalent adduct formation by metabolically activated reactive intermediates (epoxide) with nucleic acids, which could lead to mutations in the host genome [4,5,6]. Aflatoxin M1 is a hydroxylated metabolite of AFB1, converted by the liver enzymes of lactating animals that have ingested contaminated feed. This molecule maintains the same hazards as B1, belonging to Group 2B3, and showed a high stability during heat treatments (i.e., milk pasteurization).

Due to harmful effects on human and animal health, many countries have set maximum limits for mycotoxins contamination in food and feed. Regarding AFM1, the European Community has set a maximum contamination level of 50 ng/L (50 ppt) in raw milk, heat-treated milk, and milk for the manufacture of milk-based products [7]. This limit was further reduced to 25 ng/kg in infant formulae and follow-on formulae, and more in general, in dietary foods for special medical purposes intended specifically for infants [7,8]. Additionally, in the U.S., the Food and Drug Administration (FDA) established an action level for aflatoxin M1 in milk of 500 ng/L (500 ppt), as reported in the Compliance Policy Guide 527.400.

To effectively monitor a low-level occurrence of aflatoxin food contamination, sensitive and reliable diagnostic techniques are required. Currently, High Performance Liquid Chromatography (HPLC) or Liquid Chromatography–Mass Spectrometry (LC-MS) and Enzyme-Linked Immunosorbent Assay (ELISA) are the most common and official methods for mycotoxins detection in foods and feed [9]. Both techniques are closely tied to high-quality antibodies that interact specifically with the toxin of interest. The ELISA implies the use of antibodies, while the HPLC-MS analysis often requires a preliminary purification of samples by immunoaffinity columns (IAC), which concentrates the toxin, removing many contaminants of the food matrix.

Although chromatographic methods are robust techniques, immunodiagnostic assays offer advantages besides high sensitivity and selectivity such as a high throughput, a simple and fast sample preparation or no sample preparation at all, a good cost effectiveness, and they do not require a well-equipped laboratory with skilled technicians, even if mycotoxins are small molecules (<1000 Da), which necessarily require the development of a competitive ELISA (cELISA) to be detected and quantified, making the implementation of a good diagnostic more challenging.

With the aim to develop an assay exploiting an innovative technology, in this work we have isolated anti-AFM1 highly specific monoclonal antibodies, and the most promising antibody has been expressed in an alternative and cost-effective production system, such as plants. Plant cells are able to successfully express complex molecules such as antibodies of different formats, from the simplest single chain (30 kDa) [10] to the multimeric immunoglobulin A (300 kDa) [11,12]. This biotechnological approach can free the production of antibodies from the classic and more expensive systems based on animal cell cultures, which require dedicated structures and environments, specific reagents, and instruments for their growth in sterility (i.e., bioreactors/incubators). Plant molecular farming (PMF), on the contrary, allows to operate in non-sterile conditions with minimized costs (greenhouse, water, light, soil) [13]. In addition, to maximize production yields of proteins, transient expression systems (virus or *Agrobacterium*-based) rather than stable transgenic systems have been successfully optimized [14]. In this work, the agroinfiltration technique was used, which involves the infiltration of a suspension of *Agrobacterium tumefaciens*, harboring the genetic information of interest, into the plant tissues by injection with a syringe or by vacuum chamber [15,16]. Besides a high yield, this process is advantageous for its rapidity since it requires just growing cryo-conserved Agrobacteria (1–2 days), infiltrating them in plants and, after 1 week, collecting the leaf tissues for their storage or processing. Therefore, the production pipeline can be easily stopped and restarted without waste of time and materials, and even the production scale-up is immediate and inexpensive, if compared with in vitro cell cultures, simply requiring an expansion of the cultivation space dedicated to plants.

So far, many recombinant proteins have been expressed by PMF; most of these are pharmaceuticals intended for vaccine development or immunotherapy, and some of these are in clinical trials [17].

For the first time in our work, the development and validation of an efficient assay to detect aflatoxin M1 in raw milk based on a novel monoclonal antibody produced in plants is reported.

## 2. Results

### 2.1. Isolation and Characterization of Anti-Aflatoxin mAbs

Monoclonal antibodies highly specific for aflatoxin M1 were produced by hybridoma technology immunizing mice with aflatoxin M1 conjugated to ovalbumin. The cells obtained from the fusion were seeded in more than 900 wells and screened for the specificity of secreted antibodies to aflatoxin M1 by indirect ELISA in which a different conjugate (AFM1 linked to BSA) was used as the antigen. The alternation of carriers has allowed to select clones producing antibodies specific for the toxin. Approximately 5% of the wells showed specificity to aflatoxin M1, and the cells were further tested and expanded. Out of these, two clones showed a higher growing rate, secreting antibodies with higher affinity for aflatoxin M1; then, the monoclonal cell lines were isolated by limiting dilution and related antibodies were further characterized. The selected mAbs, named 5H3 and 2G3, are of different isotypes, IgG1 and IgG2b, respectively, while both possess a lambda light chain. MAbs have been purified from hybridoma cell culture medium by protein G chromatography. Their affinity to aflatoxin M1 was determined by indirect competitive ELISA and estimated in the picomolar range (Figure S1) [18]. In particular, mAb 5H3 showed a greater and more impressive affinity for the toxin (with an estimated KD = 1.88 ± 0.61 × 10^−11^ M) if compared to mAb 2G3 (KD = 1.06 ± 0.25 × 10^−10^ M).

The cross-reactivity of the two monoclonal antibodies with respect to the structurally related isoforms of aflatoxin (M2, B1, B2, G1, and G2) was also evaluated. Table 1 shows the cross-reactivity estimated as the ratio between the concentration of aflatoxin M1 that inhibits at 50% the binding of the mAb in the indirect competitive ELISA (IC_50_) with respect to the concentration observed for the other aflatoxins (%). Both antibodies showed a high selectivity for aflatoxin M1. Based on higher affinity and lower cross-reactivity for the other aflatoxins, the mAb 5H3 was chosen to develop a new assay for AFM1 detection and for being produced in plants.

### 2.2. Plant Expression of mAb 5H3

**Figure 1:**
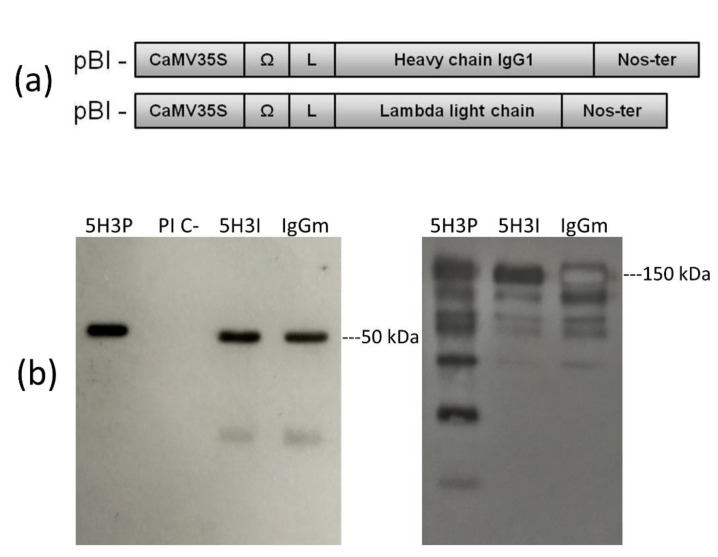
Expression of 5H3 mAb in *N. benthamiana* plant. (**a**) Schematic cassette representation of recombinant mAb constructs for plant expression. CaMV355, Cauliflower Mosaic Virus 35S promoter; Ω, translational enhancer sequence of Tobacco Mosaic Virus; Nos-ter, Nopaline synthetase terminator sequence; L, signal peptide sequence derived from an embryonic immunoglobulin. (**b**) Western blot analysis in reducing (**left**) and non-reducing (**right**) conditions of extracts (5 µg of total soluble proteins) from agroinfiltrated plant with pBIHC and LC (5H3P) or empty vector (PI C-), 50 ng of mAb 5H3 purified from hybridoma (5H3I), and 100 ng of IgG from mouse serum (IgGm). Abs were detected with HRP-labeled goat anti-mouse IgG (γ).

The genes coding for the heavy (HC) and light chains (LC) of the anti-aflatoxin M1 mAb 5H3 cloned from the hybridoma cDNA were separately inserted in a vector for expression in plants (Figure 1a). The corresponding pBI-H5H3 and pBI-L5H3 were used to transform *Agrobacterium tumefaciens* for the subsequent agroinfiltration.

To obtain the expression and assembly of the complete recombinant IgG molecule, *Nicotiana benthamiana* plants were co-infiltrated with a suspension of *Agrobacterium* harboring pBI-H5H3 and pBI-L5H3 constructs mixed 1:1 (Figure 1a). This suspension was forced to enter the leaf tissues by a syringe or using a vacuum chamber; in the latter case the plants were immersed in the *Agrobacterium* solution. After a few days (4–7 days), IgG was produced by plant cells accumulating in the leaf tissue [15].

The expression and correct assembly of the recombinant mAb 5H3 was verified by Western blot analysis of syringe-infiltrated leaf tissues collected at 6 days post infiltration (dpi) (Figure 1b). The immunoblots, performed in reducing conditions, showed bands at the expected molecular weight (50 kDa) for the recombinant IgG1 heavy chain (Figure 1a), whereas the analysis performed in non-reducing condition showed the correct assembly of the complete IgG1 with an expected molecular weight of 150 kDa (Figure 1b). Besides the 150 kDa band, some additional bands were also detected, probably corresponding to unassembled or partially assembled HC, rather than to degradation products.

The Ab accumulation in agroinfiltrated leaves was evaluated by DAS ELISA, with a maximum yield of ca. 1.6 g/kg at 6 dpi.

**Figure 2:**
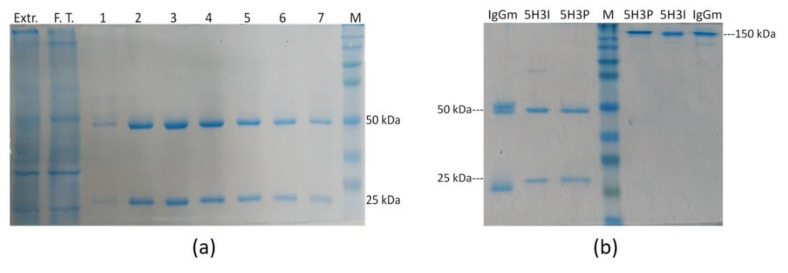
Purification of antiAFM1 antibody produced in plant. (**a**) Analysis of antibody purification by protein G-based affinity chromatography from extracts of agro-infiltrated *N. benthamiana* leaves. TSP of plant extract (Extr.), flow-through (F.T.) and fractions (1–7) eluted from protein G column were loaded on 10% SDS polyacrylamide gel under reducing conditions and stained with colloidal Coomassie blue. (**b**) Comparison between the mAb produced and purified from plant (5H3P) from hybridoma (5H3I) and mouse IgG (IgGm) under reducing (left part of the panel) and non-reducing (right part of the panel) conditions. M: Molecular Weight Marker 225,000–12,000 Da.

The recombinant mAb was purified by protein G affinity chromatography from leaves of vacuum-agroinfiltrated plants harvested 6 days post-infiltration (Figure 2a). The quality of recombinant purified mAb 5H3 was evaluated by reducing and not reducing SDS–PAGE (Figure 2b), confirming an efficient purification yield and high purity degree (>95%). SDS-PAGE under reducing conditions of eluted fractions showed the expected bands migrating at 50 and 25 kDa corresponding to HC and LC, respectively. No additional bands were detected, indicating the integrity of the purified antibody.

Finally, the functionality of the plant-produced mAb 5H3 was evaluated by ELISA and the recombinant antibody showed to retain the same specificity for aflatoxin M1 of the corresponding mAb produced in mammalian cell culture (data not shown).

### 2.3. Development of a Competitive ELISA for AFM1 Detection Using Plant-Produced mAb

A new green cELISA based on plant-derived recombinant antibody was designed by directly immobilizing the plant-produced 5H3 mAb on the plastic surface of a microtiter plate. The design of the ELISA included a pre-incubation step of the antibody with the sample containing the free toxin and a subsequent competition step by adding an AFM1-HRP conjugate (see Section 5.9).

The sensitivity of a competitive ELISA strongly depends on the concentrations of the specific mAb and of the competitor (antigen conjugated to the enzyme). Therefore, the assay was optimized by varying the concentrations of the recombinant mAb 5H3 and AFM1-HRP conjugate, according to the checkerboard titration approach. Preliminarily, the most performing antibody/AFM1-HRP ratio was defined by comparing dose–response curves obtained using different quantities of mAb (5, 10, 20, 40 ng/well) and AFM1-HRP dilutions (from 1:5000 to 1:40,000 *v*/*v*). The higher absorbance signal correlated to the minimum amount of coated mAb was obtained using 10 ng/well of antibody and a 1:15,000 (*v*/*v*) dilution of AFM1-HRP conjugate.

Additionally, the incubation times were investigated by comparing dose–response curves, specifically in relation to the pre-incubation step between the mAb and aflatoxin M1. The pre-incubation step was varied from 15 min to 1 h, keeping the incubation times of the subsequent steps as short as possible compatible with reaching a maximum signal above 1.5 O.D (15 min each). Highest sensitivity and repeatability (CV%) were achieved with 45 min of pre-incubation (data not shown). Finally, all the reaction steps were carried out at RT and required 75 min in total, divided as follows: (i) 45 min—pre-incubation of the plant-produced mAb with the free toxin, (ii) 15 min—competition with the aflatoxin M1-HRP conjugate, and (iii) 15 min—enzymatic reaction and color development.

Lastly, a reference standard curve for aflatoxin M1 quantification, using calibrators in the range from 5 to 150 ng/L, was prepared by diluting the toxin in a buffer (buffer M) designed to mimic the interference of the milk matrix and used in commercial ELISA kits for measuring AFM1. Milk is known to interfere in ELISA, so sample dilution is required to overcome the problem [19,20]. However, the dilution of the sample reduces the sensitivity of the analytical method, which is a major drawback, especially for the determination of AFM1.

**Figure 3:**
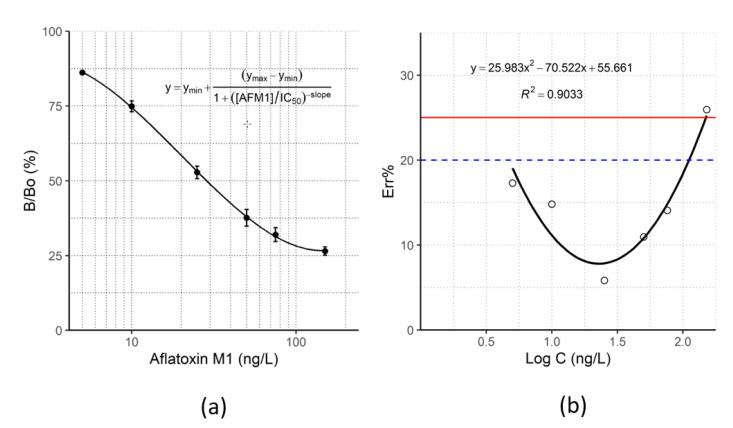
(**a**) Typical inhibition curve, obtained under optimized conditions for the development of the new green ELISA, by competition with aflatoxin M1 calibrators from 5 to 150 ng/L. Error bars show standard deviation (*n* = 5). (**b**) Accuracy of AFM1 quantification obtained with the new green cELISA. The accuracy was obtained for each calibration standard and plotted towards the AFM1 concentration. The error curve was produced by fitting with a quadratic function and used to estimate the limit of detection (LOD) and dynamic range of the assay (LLOQ–ULOQ). Blue dashed line: accuracy was considered acceptable if Err% < 20%. Definitions: Limit of Detection (LOD): Err% = 25%; Lower Limit of Quantification (LLOQ): Err% = 20%; Upper Limit of Quantification (ULOQ): Err% = 20%.

The selected experimental conditions resulted in a maximum absorbance signal > 2.0 O.D. (B_0_), and 50% inhibitor concentration (IC_50_) close to 25 ng/L. Figure 3a shows a typical standard curve obtained by competition with aflatoxin M1 calibrators from 5 to 150 ng/L.

To evaluate the proneness of the green cELISA to matrix effect, in comparison to traditional ELISA, two certified reference materials (MI 1860-1/CM and MI 1960A-1/CM) with AFM1 concentrations < 10 ng/L and 49.68 ng/L, respectively, were analyzed in four replicates on five non-consecutive days. The relative inaccuracy was calculated from the difference between the measured and the reference AFM1 values divided by the reference value (%). Regarding the Control Material with an assigned value of aflatoxin M1 < 10 ng/L, all the tested samples (*n* = 20) were correctly attributed by the new green cELISA, which provided signals comparable to the signal of the calibrator “0” (B_0_) ± 3 standard deviations of the B_0_. Regarding the Control Material naturally contaminated by 49.68 ng/L of aflatoxin M1, the mean measured value was 53.35 ± 6.87 ng/L (*n* = 20), resulting in 7.4% relative inaccuracy. The intra- and inter-assay inaccuracy was comprised within 0.9 and 13.2% with coefficient of variations below 19%.

### 2.4. cELISA Analytical Performance

The evaluation of analytical performance of the ELISA was conducted according to a strategy proposed for chromatographic methods of validation [21] and adapted to enzyme immunoassay [22]. A set of experiments was conducted as summarized in Table S1: the six matrix-matched calibrators were measured in triplicate on each day for three non-consecutive days. Inaccuracy was calculated as the difference between the estimated and the nominal AFM1 concentration for each calibrator (except for the blank) divided by the nominal AFM1 concentration (×100). AFM1 estimation was obtained by back-calculation from the dataset of replicates. In details, intra-assay inaccuracy was calculated as the mean of values obtained using two out of the three replicates to build the calibration curve and the third as an unknown sample. The permutation of replicates at each level and the repetition for three days provided 54 AFM1 back-calculated concentrations (3 values × 6 levels × 3 days). Inter-day inaccuracy was estimated on the calibration curve obtained by averaging all repeated measurements for each AFM1 level. AFM1 concentrations were estimated from the mean of replicates on each day, and the average of the daily mean values on three days (6 levels × 3 days) was compared to the nominal concentration. The coefficients of variation of the same two datasets were used to calculate intra- and inter-day (between- and within-assay) imprecision.

The analyte’s Intra- and inter-day precision fulfilled requirements for validation, as the average over all calibration levels was below 30%. Optimal precision (within ± 15% for both intra and inter-assay) was achieved in correspondence of the decision levels (25 and 50 ng/L) (Table 2). Larger variability was observed for the 150 ng/L calibrator; however, we decided to include it in the calibration curve to enable an accurate estimation of the background signal in the 4-PL equation.

Accuracy was considered optimal if the bias was below 15% and acceptable if it fell between 15% and 20%. Optimal accuracy was achieved in correspondence of the relevant levels according to European regulations (25 and 50 ng/L) and the assay enabled AFM1 quantification in the range 5–110 ng/L (bias below 20%) (Figure 3b).

The LOD, LLOQ, and ULOQ were calculated from the error curve (Figure 3b), as the level of AFM1 that can be estimated with intra- and inter-assay error below 25 and 20%, respectively, and resulted to be 3 ng/L, 5 ng/L, and 110 ng/L, respectively. In summary, the assay achieved high sensitivity and accuracy.

Finally, stability of the wells functionalized by adsorbing the plant-produced mAb was verified for 12 months at 4 °C.

### 2.5. Analysis of Spiked Raw Milk Samples

The capability of the plant-produced mAb 5H3 to detect aflatoxin M1 was further tested on raw milk samples. For this purpose, recovery experiments were carried out by fortifying a sample of raw milk with three levels of AFM1 (25, 50, and 75 ng/L) corresponding to low, medium, and high AFM1 concentrations distributed across the quantification range. The raw milk and fortified samples were analyzed without pre-treatment and after centrifugation to eliminate fatty components. Each sample was analyzed in quadruplicate and the AFM1 was estimated from the calibration curve fitted by the 4-PL equation (Table 3). The sample of raw milk was tested as containing undetectable levels of AFM1 (AFM1 below the LOD of the ELISA) for both treatments. Slight underestimation of the AFM1 amount was found for the non-treated raw milk (recovery rates: 74–87%), while satisfactory performance was achieved for the samples that underwent centrifugation to eliminate fats (recovery rates: 88–104%). Noticeably, the coefficients of variation were comparable to those measured for the calibrators, so further confirming that the matrix composition of the raw milk did not affect the assay both in terms of accuracy and precision.

## 3. Discussion

Two monoclonal antibodies highly specific for aflatoxin M1 have been isolated by hybridoma technology immunizing mice with aflatoxin M1 conjugated to ovalbumin. Both showed high affinity and were considered as ideal candidate bioreagents for developing immunoanalytical methods for determining AFM1, especially considering the stringent legal limits imposed by the European community for the quantification of this aflatoxin in milk (50 ng/L) [7].

Both antibodies showed a high selectivity for aflatoxin M1 towards other aflatoxins analyzed when compared to typical cross-reactivity reported in the literature for anti-AFM1 mAb [19,23]. In fact, both mAb 5H3 and mAb 2G3 showed low cross-reactivity (<2%) for the isoforms of fungal origin (B1, B2, G1, and G2), which are typically not found in milk, but, above all, a very low cross-reactivity for aflatoxin M2 (<1%). Although aflatoxin M2 (AFM2), a metabolite of the aflatoxin B2 (AFB2), can be found in milk and other dairy products [24], no limits are set for this toxin; however, it is preferable to have antibodies not able to recognize this isoform in the diagnostic assays, to avoid affecting the quantification of M1. Therefore, based on its greater affinity for aflatoxin M1 and a general lower cross-reactivity for the other aflatoxins, the mAb 5H3 was chosen for the subsequent steps aimed at preparing a new diagnostic immunoassay based on a plant-derived recombinant antibody.

The mAb 5H3 was expressed in plants, that allow rapid scale-up and reduction of costs in alternative to classical bioreactors [17,25]. Antibodies are complex biomolecules that cannot be synthesized in vitro, making them the most expensive reagents of an immunoassay; therefore, easy and economical systems for their production still represent a major challenge. Moreover, we exploited a transient system based on agroinfiltration technique rather than plant stable transformation, which is a very long and laborious process and results in a lower yield of recombinant protein [26]. The expression and correct assembly of the recombinant mAb 5H3 was confirmed by Western blot analysis.

The yield of plant-produced mAb after 6 days post infiltration reached ca. 1.6 g/kg, which was comparable or even better than those previously described for transiently expressed IgG [27,28,29]. The advantage of the production system is supported by possible further improvement, such as for example the use of plant virus-based systems. These have been shown to achieve extraordinary expression levels by transient expression (up to g/kg fresh weight [30,31]). Besides high expression levels, also the quality of the recombinant mAb purified from *N. benthamiana* leaves has been satisfactory. In fact, the SDS-PAGE analysis showed the integrity of the purified antibody, which therefore did not undergo degradation by plant proteases, that could affect the recombinant protein accumulation, as described in other cases [32]. Furthermore, it was possible to keep the purified mAb 5H3 for more than 12 months at 4° C without observing precipitation and/or degradation, suggesting a good stability of our plant-produced antibody.

The plant-produced, “green” mAb was employed to set up an enzyme immunoassay for measuring AFM1 in milk. Due to the sensitivity requirement (ng/L) for AFM1 determination, very efficient bioreagents should be produced to reach the goal. Especially, the specific recognition element should have high affinity, good selectivity, and specificity to be unaffected by matrix interference and long-term stability. Therefore, the performance of the newly produced, green mAb were tested for this application, as a “stress test” to confirm the potential of the plant-farming technique for producing high affinity and robust monoclonal antibodies.

Several commercial assays, exploiting conventional antibodies, exist for AFM1 quantification in milk, with performance generally adequate for assessing the presence of the toxin at the levels required by regulations [23,33]. Therefore, it was possible to directly compare the performance of the enzyme assay based on the green mAb with those of kits already existing and to investigate comprehensively the advantages and limitation of the new bioreagent.

Therefore, a conventional enzyme immunoassay was developed, in which the green mAb was anchored to wells of a standard microplate and the analyte and the tracer (AFM1 covalently linked to HRP) competed for the binding to the antibody. A sequential addition of the sample and the AFM1-HRP solutions, with a washing step interposed, was chosen to limit the interference of the milk matrix on the assay, due to the interaction with the tracer. The various steps were optimized as far as the concentrations of the reagents and time of incubation were concerned, following typical strategies for ELISA development. The optimal assay was carried out at RT and was completed in 75 min. The so-prepared wells were verified for the stability and reproducibility of the coating by replicating the calibration curve on different days over a period of 12 months and by checking coefficient of variations of replicate measurements of the same calibrator. The adhesion of the mAb 5H3 to wells was confirmed to be stable and reproducible.

As discussed above, the components of the milk matrix generally affect enzyme immunoassay performance. The interference has been variously addressed. For example, in the works of Vdoyenko et al. and Peng et al., the samples were centrifuged at high speed and diluted 10 times [19,20], thus determining a reduction in the sensitivity of the test as well as introducing a further step for sample preparation. With the aim of achieving accurate AFM1 quantification in milk, without recurring to sample dilution or pre-treatment, we used a buffer especially designed for mimicking the milk composition to preparing AFM1 calibrators. The optimized calibration curve (Figure 3a) showed satisfactory figures of merit (B0 > 2 O.D., IC_50_ ca. 25 ng/L, CV% of replicates below 20%) thus suggesting that the green mAb could efficiently replace conventional monoclonal antibodies as bioreagents for immunoassay development.

The preliminary assessment of assay performance carried out by measuring AFM1 in two certified reference materials further supported the conclusion. These results demonstrate a correct quantification of the toxin, thus suggesting that the plant-produced mAb was minimally affected by the milk matrix, or, at least, it was affected in a similar way as traditional antibodies. We concluded that the mAb 5H3 was an efficient bioreagent for enzyme immunoassay development and that the developed ELISA was able to accurately quantify the toxin in milk, without requiring additional sample treatment or dilution.

Therefore, we evaluated the analytical performance of the cELISA exploiting the plant-produced mAb. The assessment was conducted according to a strategy proposed for chromatographic methods validation [21] and adapted to enzyme immunoassay [22]. This approach relies on the execution of a limited number of experiments, which allow calculating several validation parameters, including intra- and inter-assay imprecision, inaccuracy, LOD, and range of quantification. Matrix effect was preliminary excluded as described above.

As far as LOD and the quantification range are concerned, several approaches are used to estimate these figures of merits for competitive immunoassays. Most frequently, the blank minus three standard deviation of the blank is used to calculate the limit of detection [34,35]. Alternatively, the analyte amount that inhibits the maximum signal by a defined percentage (90% for estimating LOD and 80–20% for defining the quantification range) is used [36,37]. However, to apply the first definition, enough truly blank samples are required, which are hardly available when AFM1 detection is considered. This is due to the spread of aflatoxin contamination, to the practice of mixing milk belonging to different sources and, to the poor availability of analytical confirmatory methods with sufficient accuracy to reliably measuring AFM1 below 10 ng/L. The use of defined inhibition percentage is questionable, as the accuracy and precision of different assays may vary largely. Therefore, we opted for calculating the LOD, LLOQ, and ULOQ according to the ability of the assay to provide results with an acceptable overall error [22,38]. The analytical figures of merit were satisfactory, with a LOD as low as 3 ng/L and a quantification range comprising 5–110 ng/L, which fulfill requirements for the application of the assay for measuring AFM1 at the levels relevant for European regulations.

Recovery experiments, conducted by fortifying a raw milk sample with known levels of the toxin, also confirmed the quality of the enzyme assay based on the plant-produced mAb. The advisability of using this kind of bioreagents is supported by the substantial equivalence of the performance obtained in this work, compared to those reported for similar enzyme assays, which has been summarized in Table 4 [19,23,33,39]. Even, the new green cELISA stands out for sensitivity (LOD/LOQ), analysis time, and recoveries of the toxin.

## 4. Conclusions

In this work, a new monoclonal antibody highly specific for aflatoxin M1 has been successfully selected from murine hybridoma, transiently expressed, and produced in *N. benthamiana* plants, and used to develop an innovative green competitive ELISA for the quantification of aflatoxin M1. Moreover, the new diagnostic tool for AFM1 quantification showed excellent analytical performance, achieving optimal accuracy in correspondence of the decision levels (25 and 50 ng/L), and obtaining satisfactory recovery rates of AFM1 from raw milk samples (88–104%) and Control Materials (107%). These results confirmed that the new plant-produced antibody can be a convenient substitute for in vitro-produced monoclonal antibodies for developing reliable assays, with advantages in terms of saving time and costs.

Green biotechnologies offer new cost-effective solutions such as the production of recombinant antibodies in plant instead of animal-based systems, and this is particularly true for the development of diagnostics reagents, that do not require undergoing a stringent regulatory authorization.

Immunodiagnostics, allowing the detection of very low quantities of targets of interest, still plays a major role in both the biomedical and agri-food sectors. In the latter case, a reduction of food analysis costs could consequently increase the number of tests carried out, reducing the risk of consumer exposure to harmful substances such as AFM1, which affects the entire milk supply chain.

In conclusion, our results confirm and reinforce the idea of the plant as an alternative and low-cost system to produce complex biomolecules, proposing an efficient and ready-to-use diagnostic assay for the quantification of aflatoxin M1 in milk, based on a plant-produced recombinant mAb.

## 5. Materials and Methods

### 5.1. Reagents and Apparatus

The Aflatoxin M1, M2, B1, B2, G1, and G2 analytical standard solution in acetonitrile, ovalbumin (OVA), aflatoxin B1-BSA conjugate (AFM1-BSA), complete Freund’s adjuvant (CFA), incomplete Freund’s adjuvant (IFA), RPMI 1640 medium with stable L-Glutamine, HAT Media Supplement (50×) Hybri-Max™ (hypoxanthine, aminopterin, thymidine), and mouse IgGs (I8765) were purchased from Sigma-Aldrich (Merck, Darmstadt, Germany). Penicillin and streptomycin were purchased from Euroclone (Milan, Italy). Fetal bovine serum HyClone™ (FBS), Sephadex™ G-25, HiTrap™ Protein G HP columns were purchased from Cytiva (Merck, Darmstadt, Germany). Polyethylene glycol (PEG 1500) by Roche, Merck, Darmstadt, Germany. Immobilon-P, PVDF (PVDF), Immobilon Western chemiluminescent HRP substrate were purchased from Millipore (Merck, Darmstadt, Germany). The horseradish peroxidase (HRP) was supplied by Roche CustomBiotech (Basel, Switzerland). Aflatoxin M1 powder was purchased by Fermentek Ltd. (Jerusalem, Israel). HRP-labeled goat anti-mouse IgG (γ) (KPL, 474–1802, LGC Clinical Diagnostics, from SeraCare Life Science, Milford, MA, USA). Rneasy Plant Mini Kit (QIAGEN, Hilden, Germany). Mouse MonoAb-ID Kit (HRP) isotyping test kit, microtiter plates Nunc Maxi-Sorp™, 1-Step™ Ultra TMB (3,3′,5,5′-tetramethylbenzidine)-ELISA Substrate Solution (TMB), SuperScript™ II Reverse Transcriptase and accuPrime™ Pfx DNAPolymerase (Invitrogen) were purchased from Thermo Fisher Scientific (Waltham, MA, USA).

The buffer mimicking milk composition (buffer M) was kindly provided by Euroclone S.p.A. (Milan, Italy).

The Control Materials MI1960-1/CM (lyophilized, partially defatted, raw bovine milk incurred with Aflatoxin M1: assigned value 49.68 ng/L) and MI1860-1/CM (assigned value < 10 ng/L) were purchased from Test Veritas S.r.l. (Trieste, Italy).

A microtiter plate reader (Sunrise™, TECAN, Männedorf, Switzerland), and TE70 Semi-Dry Transfer Unit (Cytiva, Merck, Darmstadt, Germany) were also used.

### 5.2. Conjugates’ Preparation

Aflatoxin M1 was linked to ovalbumin (AFM1-OVA) and horseradish peroxidase (AFM1-HRP), as reported previously [40]. Briefly, aflatoxin M1-O-(carboxymethyl)oxime was linked to proteins by the DCC/NHS ester method in a molar ratio of 50:1 and 10:1 (hapten to OVA or HRP, respectively). The separation of the conjugate from by-products and excess of the hapten was carried out by gel filtration on a Sephadex™ G-25 cartridge (mobile phase: phosphate buffer saline). To calculate the modification index (AFM1 residues per protein molecule), the AFM1 of the conjugate was estimated by UV-vis spectrophotometry, by considering the specific absorption peak of AFM1 at 357 nm and employing an extinction coefficient for AFM1 in PBS of 3411 M^−1^ cm^−1^. The OVA amount was quantified by the Brilliant Blue Comassie method while HRP amount was calculated from the specific absorbance at 402 nm.

### 5.3. Mice Immunization and Blood Sampling

Four 9-week-old mice were immunized intraperitoneally with 40 µg of the immunogen AFM1-OVA conjugate in 0.1 mL of PBS that has been emulsified with an equal volume of CFA immediately prior to injection. A booster injection, at 40 µg of AFM1-OVA conjugate in 0.1 mL of PBS, has been emulsified with an equal volume of IFA, and was made 2 weeks after the first injection. Lastly, a second booster injection of 40 µg of AFM1-OVA in 0.1 mL of PBS was administered after 5 weeks in the tail vein. All experiments were performed according to the Directive 2010/63/EU, approved by the local Ethical Committee for Animal Experiments of the ENEA, and authorized by the Italian Ministry of Health (n◦ 721/2016-PR issued on 22/07/2016).

Blood samples (approximately 0.1 mL of blood from the tail vein) were collected before mice immunization and 5 days after the first boost injection. Sera were prepared by centrifugation (1000× *g*, 10 min) of the coagulated blood and stored as aliquots at −20 °C until further use. An indirect ELISA, described below, was used to determine the serum antibody titer.

Cell preparations and culture conditions. Myeloma cells (P3X63Ag8.653) were cultured in RPMI supplemented with 20% FBS (*v*/*v*) and antibiotics (100 U/mL penicillin + 100 mg/L streptomycin) under standard humidified conditions (37 °C, 95% H_2_O, 5% CO_2_). Revertants were eliminated via 8-azaguanine treatment (20 µg/mL, three medium changes). For the fusion process, myeloma cells were seeded at a density of 1 × 10^5^ cells/mL in culture flasks and allowed to proliferate for four days resulting in a high yield of viable cells in logarithmic growth phase.

B-lymphocytes from the spleens of immunized mice were freshly isolated (1.39 × 10^8^) and fused with myeloma cells (1.88 × 10^7^) using 50% aqueous hydrophilic PEG 1500, according to manufacturer’s recommendations.

After the fusion procedure, cells were seeded (50 µL/well) into 10 96-well plates. For hybridoma cells selection, HAT supplement treatment was used. To ensure monoclonality, single cells producing the desired antibody were isolated from the mixture by limiting dilution cloning. Hybridoma clones, which tested positive for antibody production, were cloned again and then further expanded. Cell lines were cryopreserved and stored in liquid nitrogen.

### 5.4. Monoclonal Antibodies Characterization

Indirect ELISA. This ELISA was used to screen for specific antibodies produced by hybridoma cells and to evaluate Ab accumulation in agroinfiltrated leaves. Hybridoma culture supernatants were collected four days after the last medium change. Microtiter plates were coated with 100 µL/well AFM1-BSA (2 ng/µL) in PBS, overnight at 4 °C to set up the solid phase. The screening was performed using a different protein carrier to eliminate the unspecific signal due to the preponderant presence of antibodies directed to OVA. Plates were washed with 3 × 300 µL washing buffer (PBS with 0.05% (*v*/*v*) Tween 20) per well and blocked for 2 h at 37 °C with 250 µL blocking buffer (2% (*w*/*v*) no fat milk in PBS). Then, 100 µL sample/well was incubated for 1 h at 37 °C. Plates were washed again and 100 µL/well of HRP-labeled goat anti-mouse IgG (γ) diluted 1:5000 in 0.2% no fat milk (*w*/*v*) in PBS were applied and incubated for 1 h at 37 °C. After additional washing steps, TMB was used as substrate. The absorbance was measured after 15 min at 620 nm and the wells featured the highest absorbance values were selected.

To evaluate (i) the detection range of the selected AFM1-antibodies, (ii) the mAbs KD (according to Friguet et al., [18]) and (iii) the cross-reactivity to other structurally related molecules, an indirect competitive ELISA was carried out using free AFM1, and AFM2, AFB1, AFB2, AFG1, and AFG2 as potential binding competitors. The relative cross-reactivity was determined as IC_50_ (AFM1)/IC_50_ (other aflatoxins) × 100. Data were plotted using the R graphics package ggplot2 [41].

RNA purification and RT-PCR. Starting from 1 × 10^6^ hybridoma cells, secreting mAbs against aflatoxin M1, the total RNA was purified using the Rneasy Plant Mini Kit, according to manufacturer’s recommendations. cDNA was synthesized using SuperScript™ II Reverse Transcriptase in a mixture of: 3 µg RNA, 2 µL (5×) reaction buffer, 500 µM dNTPs, 0.8 µM oligo (dT)12-18, 10 mM DTT, 40 U RNAse inhibitor, and dH_2_O DEPC to the final volume of 20 µL. After 5 min incubation at 65 °C, the reaction was placed on ice for 1 min and 200 U of reverse transcriptase was added. The reaction mixture was then incubated at 42 °C for 1 h and held at 72 °C for 15 min and cooled to RT.

The cDNA was amplified using accuPrime™ Pfx DNA. Each PCR reaction contained: 2 µL of cDNA, 20 pmol 5′ and 3′ primers, 250 µM dNTPs, 5 µL of reaction buffer (10×), 3 mM MgCl_2_, 2.5 U of polymerase and sterile dH_2_O up to a final reaction volume of 50 µL. Cycling conditions were: initial melt at 94 °C for 2 min followed by 30/40 cycles of a three-step program (94 °C, 1 min; 58 °C, 1 min; and 72 °C, 1 min). The reactions were then held at 72 °C for 7 min and cooled to 4 °C.

### 5.5. Gene Engineering

The genes encoding the antibody heavy and light chains (HC and LC) were amplified from the cDNA of the hybridomas using a set of degenerated primers described in the literature for HC gene and designing new primers, derived from Kabat database, for LC gene [42,43], (VLambda: 5′-CAGGCTGTTGTGACTCAGG and CLambda: 5′-CTAGGAACASTCAGCACGGGAC for LC, and MH1: 5′-SARGTNMAGCTGSAGSAGTC and Gamma_rev: 5′-TCATTTACCAGGAGAGTGGG for HC). The amplified HC and LC sequences were cloned in an intermediate vector, pBS-SK, digested with EcoRV and sequenced. Then, HC and LC genes were amplified again, using primers to insert appropriate restriction sites at the beginning and the end of sequences encoding for mature proteins (Lambda_for: 5′-GAG**CTGCAG**GTGGTACCTCGCAGGCTGTTGTGACTCAGG, Lambda_rev: 5′-TGG**CCCGGG**CTAGGAACAGTCAGCACGGGAC for LC, and IgG1_for: 5′-GAGCTGCAGGT**GGTACC**TCGGAGGTTCAGCTGGTGGAGTC, IgG1_rev: 5′-CCG**GAGCTC**TCATTTACCAGGAGAGTGGG for HC), and cloned in the vector pBI-121 for plant expression. PCRs were performed using AccuPrime™ Pfx DNA Polymerase. The sequences of all constructs were verified by Sanger sequencing (BMR Genomics, Padua, Italy).

### 5.6. Transient Gene Expression

Transient expression was performed by agroinfiltration of *N. benthamiana* plants. *Agrobacterium tumefaciens* (strain LBA 4404) transformed with pBI_H5H3, pBI_L5H3 were grown separately O/N at 28 °C, centrifuged, and suspended in 10 mM MES, pH 5.5 (each at O.D. 600 nm = 0.6). To obtain full IgG, equal volumes of suspension were mixed with a suspension of *Agrobacterium* (at 1:1:1 ratio) harboring the gene silencing suppressor p19 from TBSV, to enhance the expression levels [44]. Six-week-old *N. benthamiana* plants (at the 6–7 leaf stage), grown at 24 °C with a 16 h light and 8 h dark cycle, were infiltrated with this suspension either by using a syringe, for a preliminary evaluation of Ab expression (Western blot analysis and Ab raw yield), or vacuum, for Ab purification. In the latter case, plants were infiltrated by completely submerging each plant in the *Agrobacterium*-containing solution inside a vacuum chamber. Vacuum was applied and then quickly released. The infiltrated leaves were harvested 6 days post-infiltration and immediately processed, or frozen in liquid N_2_ and stored at −80 °C before use.

### 5.7. Antibody Purification

Batches of 20 g of plant tissue were ground in liquid nitrogen using a mortar and pestle and homogenized in PBS. The homogenate was pre-filtered through Miracloth (Millipore, Merck, Darmstadt, Germany) and centrifuged for 20 min at 40,000× *g*. The supernatant was then passed through a 0.45 µm filter and loaded on a 1 mL Protein G column after equilibration with PBS, according to the manufacturer’s recommendations. After feed injection, the column was washed with PBS buffer to remove unbound material, and the bound material was eluted with 100 mM glycine buffer, pH 2.7. The eluted samples were immediately neutralized using 1 M Tris–HCl (pH 9.0), to minimize degradation and aggregation of the antibody, and analyzed by sodium dodecyl sulphate–polyacrylamide gel electrophoresis (SDS-PAGE).

### 5.8. Western Blot Analysis

Five micrograms of total soluble proteins were resolved by 10% SDS–PAGE and transferred to PVDF using the transfer system. HRP-labeled goat anti-mouse IgG (γ) diluted 1:5000 in 2% (*w*/*v*) skim milk in PBS was used to detect HC using HRP substrate. Mouse IgGs were used as a positive control.

### 5.9. Enzyme Immunoassay Exploing the Plant-Produced mAb

Microtiter plates were coated with 200 µL/well of plant-produced mAb 5H3 in PBS, overnight at 4 °C. Plates were washed with 4 × 300 µL washing buffer (PBS with 0.05% (*v*/*v*) Tween 20) per well and blocked for 2 h at 37 °C with 300 µL blocking buffer (PBS with 1% (*w*/*v*) BSA), stabilized and sealed under vacuum. Then, 200 µL sample or AFM1 standards/well were pre-incubated for 45 min at room temperature (RT), to let the AFM1 bind to the specific antibodies of the solid phase. Plates were washed again and 200 µL/well of AFM1-HRP were dispensed and incubated for 15 min at RT. After an additional washing step, TMB was added to the wells as substrate for the HRP enzyme activity. The optical density (O.D.) was measured after 15 min, stopping the reaction with 50 µL/well HCl 3M, and reading at 450 nm via microtiter plate reader. Data were plotted using the R graphics package ggplot2 [41]. Calibration curves were interpolated by the four-parameter logistic Equation (4-PL) [45], as follows:

y = ymin + (ymax − ymin)/1 + ([AFM1]/IC_50_)^−Slope^

where ymin is the background signal estimated at infinite [AFM1], ymax is the maximum signal (which corresponds to the signal at [AFM1] = 0), IC_50_ is the AFM1 concentration that halves the ymax (50% inhibition of the maximum binding), and Slope is the slope of the curve in correspondence of IC_50_.

### 5.10. Immunoassay Analytical Assessment

The evaluation of analytical performance of the green cELISA included the evaluation of the following parameters: limit of detection (LOD), range of quantification (comprised between the lower limit of quantification, LLOQ, and the upper limit of quantification, ULOQ), accuracy, precision (within- and between-assay), and recovery.

Accuracy and precision were investigated by applying the validation strategy developed by Alladio et al. for chromatographic methods [21]. According to the strategy, six calibrators (from 5 to 150 ng/L of AFM1) prepared in the matrix-matched diluent (kindly provided by Euroclone, Milan, Italy) were analyzed in triplicate within the assay and on three different days (between-assay/between-day variability). Data were normalized by calculating B/B0 (i.e., by dividing the absorbance measured by the mean absorbance of the blank).

The set of 54 data was treated as in Alladio et al., 2020 (a schematic of the strategy is shown in Table S1) [21]. From the analysis, we obtained the within- and between-assay imprecision for each individual calibrator and the mean value for all levels (Table 3). Additionally, the inaccuracy (which was defined as: ‘calculated AFM1 value’ − ‘nominal AFM1 value’/‘nominal AFM1 value’ × 100) was obtained for each calibration level and plotted towards the AFM1 concentration. The so-obtained error curve was fitted by a quadratic function to estimate the limit of detection and dynamic range [22]. The LOD, LLOQ, and ULOQ values were defined based on the error curve, as the AFM1 level that can be measured with an acceptable inaccuracy of 25% and 20%, respectively [21].

### 5.11. Preparation of Spiked Raw Milk Samples

Raw milk samples were purchased from a local dairy. Each raw milk sample was weighted, to define the correct volume (d = 1.030 g/cm^3^), and spiked adding 25, 50, and 75 ng/L of aflatoxin M1 by adding the proper toxin volume with a Hamilton glass syringe. Afterwards, the samples were directly tested or centrifuged at 4 °C at 3000× *g* for 10 min for fat removal.

## Figures and Tables

**Table 1:** Cross-reactivity of monoclonal antibodies with respect to aflatoxins.

	Cross-Reaction (%)					
	Aflatoxins					
mAbs	M1	M2	B1	B2	G1	G2
5H3	100	<1	<0.2	<0.2	<0.2	<0.2
2G3	100	<1	<1.75	<0.2	<1.2	<0.2

**Table 2:** Imprecision of the new green cELISA for detecting AFM1.

CV%		
AFM1 (ng/L)	Within-Assay ^a^	Between Assay ^b^
5	20.3	26.7
10	12.8	24.0
25	5.7	9.7
50	14.4	10.6
75	11.9	24.9
150	36.6	62.6
Mean	17.0	26.4

^a^*n* = 9, ^b^ *n* = 3.

**Table 3:** Recovery values obtained from a sample of raw milk fortified with AFM1 and analyzed without any pre-treatment and after centrifugation (10 min at 3000× *g*, 4 °C).

AFM1 Fortification Level (ng/L)	Raw Milk (Without Centrifugation)			Raw Milk (Centrifuged)		
	Measured AFM1 ± SD ^a^ (ng/L)	Recovery (%)	CVa (%)	Measured AFM1 ± SD ^a^ (ng/L)	Recovery (%)	CV ^a^ (%)
0	nd ^b^			<LOD		
25	19.8 ± 2.1	79	11	24.6 ± 3.6	99	14
50	37.0 ± 3.2	74	9	44.1 ± 0.7	88	2
75	65.1 ± 1.7	87	3	77.7 ± 9.3	104	12

^a^*n* = 4. ^b^ Not detectable (Absorbance was higher than that provided by the calibrator 0 ng/L).

**Table 4:** Summary of the most important analytical parameters (LOD, LOQ, time, recovery%) of our green ELISA and other ELISA assays or commercial kits validated and published in the last years.

Milk Sample	Kit Validated	AFM1 Expected (ng/L)	AFM1 Measured (ng/L)	Recovery (%)	LOD-LOQ (ng/L)	IC_50_ (ng/L)	Time Required	Reference
Skimmed	-	35	-	107.6	27.5–35.0	64.7	75 min	[19]
		70	-	87.5				
		140	-	85.3				
Skimmed	-	20	17.0 ± 1.4	85.2	3–n.d. ^a^	70	135 min	[39]
		50	50.3 ± 2.8	100.6				
		200	221.8 ± 12.5	110.9				
Skimmed	I’screen AFLA M1	5	5.8	116	1.1–2.5	25	75 min	[23]
		20	19.4	97				
		50	52.1	104				
		100	103.9	104				
		200	203.7	102				
Powdered	I’screen AFLA M1	11.1	11.0	99	1.8–4.6	25	75 min	[23]
		52.82	52.5	99				
Skimmed	Agraquant	30	-	105	8.0–13.8	-	75 min	[33]
		55	-	99				
		80	-	86				
		105	-	75				
Skimmed	BioShield ES	30	-	109	7.9–14.4	-	75 min	[33]
		55	-	117				
		80	-	95				
		105	-	84				
Skimmed	Ridascreen	30	-	107	9.4–15.7	-	75 min	[33]
		55	-	117				
		80	-	101				
		105	-	82				
Skimmed	green ELISA	25	24.6 ± 3.6	99	3–5	25	75 min	This paper
		50	44.1 ± 0.7	88				
		75	77.7 ± 9.3	104				
Powdered	green ELISA	49.68	53.4 ± 6.9	107	3–5	25	75 min	This paper

^a^ Not determined.

## Data Availability

Not applicable.

## Supplementary Materials

The following supporting information can be downloaded at: https://www.mdpi.com/article/10.3390/toxins14120851/s1, Figure S1: Scatchard plot of the binding of the two mAbs; Table S1: Scheme used for the back-calculation to build the error curve.
