# Supplementary material for: Design of a Diagnostic Immunoassay for Aflatoxin M1 Based on a Plant-Produced Antibody
Source: Toxins (Basel). 2022 Dec 3;14(12):851. doi: 10.3390/toxins14120851 (PMC9781297; doi:10.3390/toxins14120851)
Supplement: Supplementary file 1 [file toxins-14-00851-s001.zip › toxins-2043357-supplementary.pdf]

toxins

MDPI

Figures

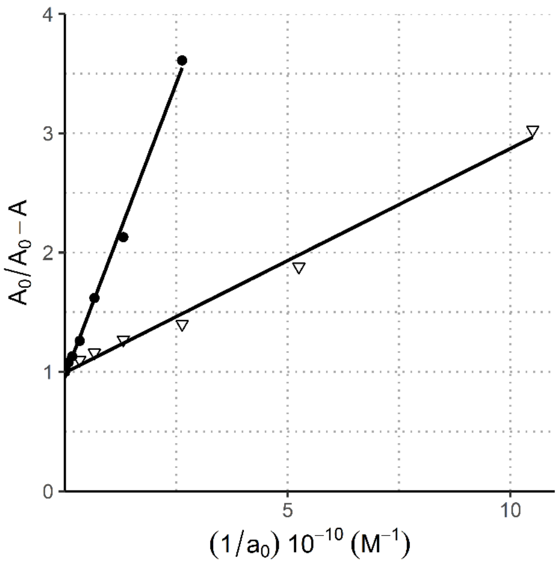

Figure S1. Scatchard plot of the binding of the two mAbs (▽, 5H3; ●, 2G3), measured by indirect competitive ELISA.

Tables

Table S1. Scheme used for the back-calculation to build the error curve.

	Intra-assay			Inter-assay		
	Calibration	'Unknown'	Result (AFM1, ng/L)	Calibration	'Unknown'	Result (AFM1, ng/L)
Day 1	Rep1,2	Rep3	Calc1_1	Rep1,2,3@day1,2,3	Rep1,2,3@day1	Calc1
	Rep1,3	Rep2	Calc2_1			
	Rep2,3	Rep1	Calc3_1			
Day 2	Rep1,2	Rep3	Calc2_1		Rep1,2,3@day2	Calc2
	Rep1,3	Rep2	Calc2_2			
	Rep2,3	Rep1	Calc2_3			
Day 3	Rep1,2	Rep3	Calc3_1		Rep1,2,3@day3	Calc3
	Rep1,3	Rep2	Calc3_2			
	Rep2,3	Rep1	Calc3_3			
			**Mean, SD, CV% (*n*=9)**			**Mean, SD, CV% (*n*=3)**

Each calibrator was measured in three replicates (Rep) on each day for three non consecutive days. The intra-assay inaccuracy was calculated by using the mean of two replicates for the calibration curve and the third as an unknown sample. The estimated concentration for the unknown samples (Calc) was divided by the nominal value of the calibrators and reported as percentage (%Err). The imprecision and inaccuracy were calcualted for each level as the mean of three replicates of bac-calculated concentrations. For the inter-assay inaccuracy, the same data were used; however, the calibration curve was obtained by considering all replicates (n = 9) and the mean of the three replicates was used as the unknown sample to obtain Calc. Again, the imprecision and inaccuracy were estimated from the mean and standard deviation of Calc values.
